# Mindfulness-based relapse prevention for drug addiction in Nepal: impact and implications of an initial 2-day training of trainers

**DOI:** 10.1192/bji.2024.37

**Published:** 2025-02

**Authors:** Arun Jha, Bharat Goit, Robin Jha, Prerna Jha, Prabhat Kiran Pradhan, Achyut Acharya

**Affiliations:** 1 Consultant Psychiatrist, Hertfordshire Partnership University NHS Trust, St Albans, UK. Email: arunjhauk@gmail.; 2 Professor of Psychiatry, National Medical College, Birgunj, Nepal; 3 Consultant Psychiatrist, Province Hospital, Janakpur, Nepal; 4 Consultant Psychiatrist, Everest Hospital, Kathmandu, Nepal; 5 Retired Mental Health Social Worker, Maryknoll, Kathmandu, Nepal; 6 Consultant Psychiatrist, BG Hospital and Research Centre, Pokhara, Nepal

**Keywords:** Addiction, relapse, prevention, mindfulness, training, Nepal

## Abstract

Drug addiction is rife in Nepal, with a high relapse rate following treatment. Apart from basic psychosocial support, there are no evidence-based aftercare services for individuals in recovery. Recently, mindfulness-based interventions have shown promising results in preventing relapse. We discuss the context, challenges and opportunities of organising a 2-day intensive face-to-face mindfulness-based training for Nepalese mental health professionals to facilitate 8-week mindfulness-based relapse prevention (MBRP). Altogether, 24 participants completed the feedback questionnaire. Most were rehabilitation staff, along with a few psychologists and psychiatrists. Feedback suggested a high degree of satisfaction and provided comments to improve the programme. It has prompted us to design online MBRP training and set up a feasibility study for an MBRP programme in Nepal. If successful, this may help a huge number of individuals in recovery.

In contrast to the traditional view of moral shortcoming, addiction is currently conceptualised as a mistaken understanding of substance use as a refuge from suffering.^1^ Craving, a significant predictor of relapse, has been added as a symptom for substance use disorder (SUD) in DSM-5.^2^ Relapse refers to return to pretreatment levels of substance use after a period of sobriety. Relapse rates in Nepal are as high as 75% during 6-month period following treatment.^3^ There are no evidence-based after-care programmes in Nepal, owing to lack of community mental health services.^4^

Research informs that mindfulness practice can address the roots of craving and reactive behaviour. Mindfulness practice allows the practitioner to directly observe the mind's behaviour, and to self-monitor thoughts and feelings with acceptance and non-judgemental objectivity. Bowen et al^5^ developed a mindfulness-based relapse prevention (MBRP) programme designed to reduce the risk and severity of relapse following substance misuse treatment. The programme involves eight weekly 2 h group sessions delivered over consecutive weeks. Sessions focus on raising awareness of environmental triggers and the physical, affective and cognitive reactions that follow, bringing awareness to the progression of reactions that occur in response to such cues. To increase ability to tolerate the discomfort associated with craving, patients maintain an ongoing practice of both formal meditation and of exercises designed to increase awareness of triggers and reactions.

## Implementing MBRP in Nepal

Trainers’ training for MBRP group facilitators is not widely available. It is non-existent on the Indian subcontinent. We adapted the MBRP manual^6^ to train professionals working in addiction care. Preparation for facilitation of MBRP involves an established personal mindfulness practice as well as formal training in the model. The background and basic theory of relapse prevention, mindfulness meditation and the blending of these practices are important components of the training in MBRP. However, the central focus is on experiential learning. We describe some issues that we encountered. Necessary modifications were made to better suit the Nepalese setting. We have tried to adhere to the underlying intention of each exercise.

### Training of trainers

To adequately guide different aspects of the MBRP programme, facilitators require experience in treatment for addiction as well as in group facilitation. The MBRP training helps facilitators develop present-moment-centred, non-judgemental and accepting qualities cultivated by mindfulness practice itself. The aim is to meet whatever arises – among facilitators and patients alike – with curiosity and compassion with an experiential focus.

Bowen and colleagues^6^ describe a 3-day workshop in which the theory and rationale are presented on the first evening, followed by 2 full days of guidance through the eight sessions of MBRP. For logistic reasons, we opted for a 2-day workshop (12 h in total) with one 4 h session on the first day and two sessions (4 h each) on the second day (Table 1).

**Table 1:** Timetable and sessions in the mindfulness-based relapse prevention (MBRP) training programme

Day 1 (16 July)	Day 2 (17 July)
Registration: 15.00–16.00 h **Evening slot**: 16.00–20.00 h (sessions 1 and 2) Dinner: 20.00–22.00 h	**Morning slot**: 08.00–12.00 h (sessions 3, 4 and 5) Lunch: 12.00–13.00 h **Afternoon slot**: 13.00–17.00 h (sessions 6, 7 and 8)

Session 1 started with the theory and rationale, followed by guidance through the eight sessions of MBRP, with several practices conducted in real time. Similar to the MBRP treatment protocol, the practices and exercises precede discussions, with a significant part of each session spent on the meditation and exercises themselves.

## Challenges and opportunities

### Challenges

Challenges experienced in implementing MBRP in Nepal include training for the trainer, identifying potential facilitators, communication and persuasion, adapting the MBRP manual in Nepalese, preparing mindfulness audios in the Nepalese language, organising a suitable training venue in Nepal, finding funding to print the manual, hall hire and subsistence for the 2-day intensive training, and coordinating the registration process, communicating with participants and organising follow-up supervision. Each challenge is discussed individually.

### Identifying potential facilitators

Our first hurdle was to find suitably qualified mental health professionals willing to learn and deliver the MBRP programme in Nepal. Most professionals were curious and superficially interested but reluctant to commit. After a series of Zoom meetings and personal telephone calls an MBRP WhatsApp group was created to launch the programme in Janakpur, a provincial town in south central Nepal.

We managed to identify volunteers from four different cities – Janakpur, Birgunj, Pokhara and Kathmandu. Two lay volunteers participated but they had difficulty understanding the concept of relapse prevention. After attending the course, they were unable to engage in personal mindful practice and were unable to identify even five potential participants to run a group.

### Registration process

One of the authors (P.K.P.) agreed to provide administrative support for inviting and communicating with the participants. All potential participants were sent a registration form to show their interest in the MBRP training programme. Registration was free, but participants were expected to arrange their own travel to and accommodation in Janakpur.

### Preparing training materials

One of the authors (A.J.) adapted the MBRP manual and prepared audio materials in Nepalese. It took over 3 months of continuous work, commencing in April 2024. Recording mindfulness exercises was daunting because it required learning, practising those exercises and sharing with participants. Recordings were made using Apple's Voice Memos app on an iPhone. Audio tracks are available at www.practicembrp.com. Translating certain words (‘mindfulness’, ‘SOBER space’, ‘urge-surfing’, etc.) into Nepalese was at times also difficult owing to unavailability of such words in the Nepalese language.

### Personal mindfulness practice

The facilitator's personal mindfulness meditation practice is crucial for facilitating MBRP groups. Feedback shared informally with the course organiser indicated that after the completion of training, most participants had started practising mindfulness, but not daily. Finding time for regular practice and poor understanding of the programme were two major constraints. Some participants had started using MBRP exercises with individual patients despite being aware of the group delivery format. This may result in a restricted ability to respond skilfully to questions, doubts and misconceptions raised by participants. This information has prompted us to offer additional online training for existing and new participants.

## Learning and feedback

In the final session of the course, participants were asked about experiences of the course and were asked to complete a feedback form (Table 2).

**Table 2:** Reflections on the mindfulness-based relapse prevention (MBRP) course: feedback questions and common responses

Question	Responses (examples)
What did you find most valuable about this course? What, if anything, did you learn?	Helpful in dealing with personal life difficulties; learned to find time for myself; learning by practising; patience and concentration for mindfulness; mindful check-in; doing things carefully
What, if anything, has changed for you during the course as a result of your participation?	It has made me easy going; peaceful; inspired to remain always mindful; check-in; I will now do things consciously; unique; should be organised regularly
Was there anything that got in the way of your learning or growth or that might have improved the course for you?	Unclear audio (external speakers); duration was a bit short; no previous exposure or knowledge of mindfulness
Other comments?	Not more than two guided meditations in one day; need regular practice
On a scale of 1 (not at all) to 10 (very), how important has this programme been to you? Please explain why you have given this rating.	Effective in daily life; very useful if incorporated in practical life; I realised how much I learned; how to live in recovery
On a scale of 1 (not at all) to 10 (very), how likely are you to continue engaging in formal mindfulness practice (e.g. body scan, sitting meditation, mindful stretching/movement) after this course?	Body scan: 10; sitting meditation 8; mindful stretching/movement: 10
On a scale of 1 (not at all) to 10 (very), how likely are you to continue engaging in informal mindfulness practice (e.g. SOBER space, mindful eating, daily activities) after this course?	SOBER space: 10; mindful eating: 7; daily activities: 8

Initially, only 19 participants had registered for the course, of which 12 were male, but 27 delegates participated and 24 completed the feedback forms.

Key lessons learned from the Janakpur training include: (a) the MBRP-Nepal programme should recruit only rehabilitation staff; (b) follow-up group supervision is required to supplement the initial training; (c) role-play should be included in each session; (d) better quality audio equipment (external speakers) should be used in future training; (e) an introductory didactic (class-type) session should be included focusing on relapse and relapse prevention.

## Discussion

Similar to the findings from a recent Brazilian study,^7^ the overall feedback of the initial intensive MBRP training for trainers in Nepal appears encouraging. Most participants found it useful and provided valuable suggestions to improve the programme. Most intend to continue practising both formal and informal mindfulness exercises. The feedback helped us modify the programme for future use. Research is underway to assess the feasibility of an online MBRP training for Nepalese professionals working in residential/in-patient detoxification settings. It remains to be seen whether the MBRP-Nepal programme makes any difference on the ground.
